# Supplementary material for: Exploring the Complexity of the Human Respiratory Virome through an In Silico Analysis of Shotgun Metagenomic Data Retrieved from Public Repositories
Source: Viruses. 2024 Jun 13;16(6):953. doi: 10.3390/v16060953 (PMC11209621; doi:10.3390/v16060953)
Supplement: Supplementary file 1 [file viruses-16-00953-s001.zip › Conradie_et_al_Supplemental/Conradie_SupplementalMaterialRevised.docx]

Supplementary Material

Exploring the complexity of the human respiratory virome through an *in-silico* analysis of shotgun metagenomic data retrieved from public repositories.

Talya Conradie^1,2^, Jose A. Caparrós-Martín^1^, Siobon Egan^2,3^, Anthony Kicic^1,4,5,6^, Sulev Koks^2,7,8^, Stephen M. Stick^3,5^ and Patricia Agudelo-Romero^1,9,10*^.

^1^Wal-yan Respiratory Research Centre, Telethon Kids Institute, Perth, WA, Australia.

^2^Medical, Molecular and Forensic Sciences, Murdoch University, Perth, WA, Australia.

^3^Centre for Computational and Systems Medicine, Health Futures Institute, Murdoch University, Perth, WA, Australia.

^4^Department of Respiratory and Sleep Medicine, Perth Children’s Hospital for Children, Perth, WA, Australia.

^5^Centre for Cell Therapy and Regenerative Medicine, School of Medicine and Pharmacology, Perth, WA, Australia.

^6^School of Population Health, Curtin University, Perth, WA, Australia

^7^Perron Institute for Neurological and Translational Science, Perth, WA, Australia.

^8^Centre for Molecular Medicine and Innovative Therapeutics, Murdoch University, Perth, WA, Australia.

^9^Australian Research Council Centre of Excellence in Plant Energy Biology, School of Molecular Sciences, The University of Western Australia, Perth, WA, Australia.

^10^European Virus Bioinformatics Center, Jena, TH, Germany.

*** Correspondence:**

Corresponding Author: Patricia Agudelo-Romero, PhD.

[Patricia.AgudeloRomero@telethonkids.org.au](mailto:Patricia.AgudeloRomero@telethonkids.org.au)

Keywords: Respiratory viruses, shotgun metagenomics, lung virome, genome assembly, viromics, microbiome, airways, omics.


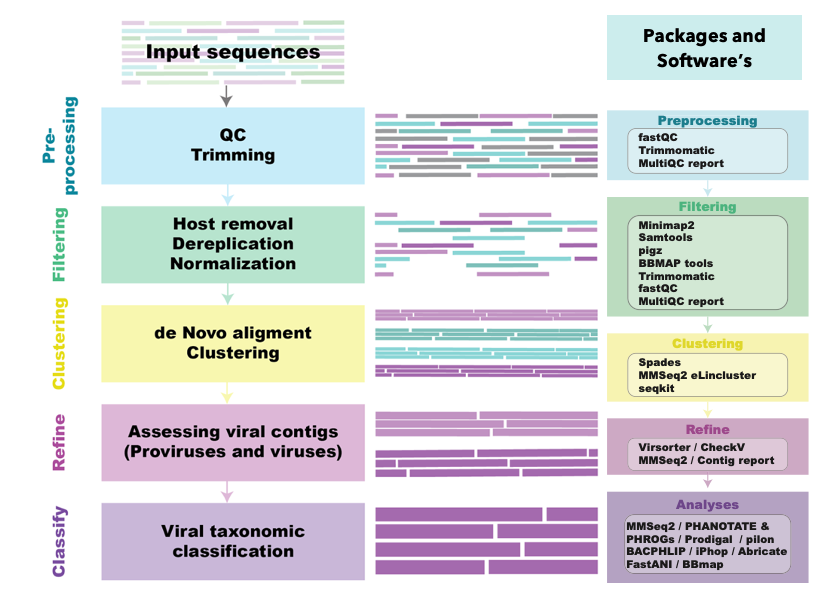


**Supplementary Figure S1**. Diagram depicting EVEREST, a pipEline for Viral assEmbly and chaRactEriSaTion, processes and software dependencies. Input fastq files undergo pre-processing quality control by sequence trimming, followed by removing host and replicated sequences, and digital normalisation. Then *de novo* sequence alignment, and sequence clustering allow for contig refinement by assessing the presence of viruses, which are taxonomically classified through nucleotide (NCBI; nucleotide) and amino acid (Uniprot; amino acids) databases.


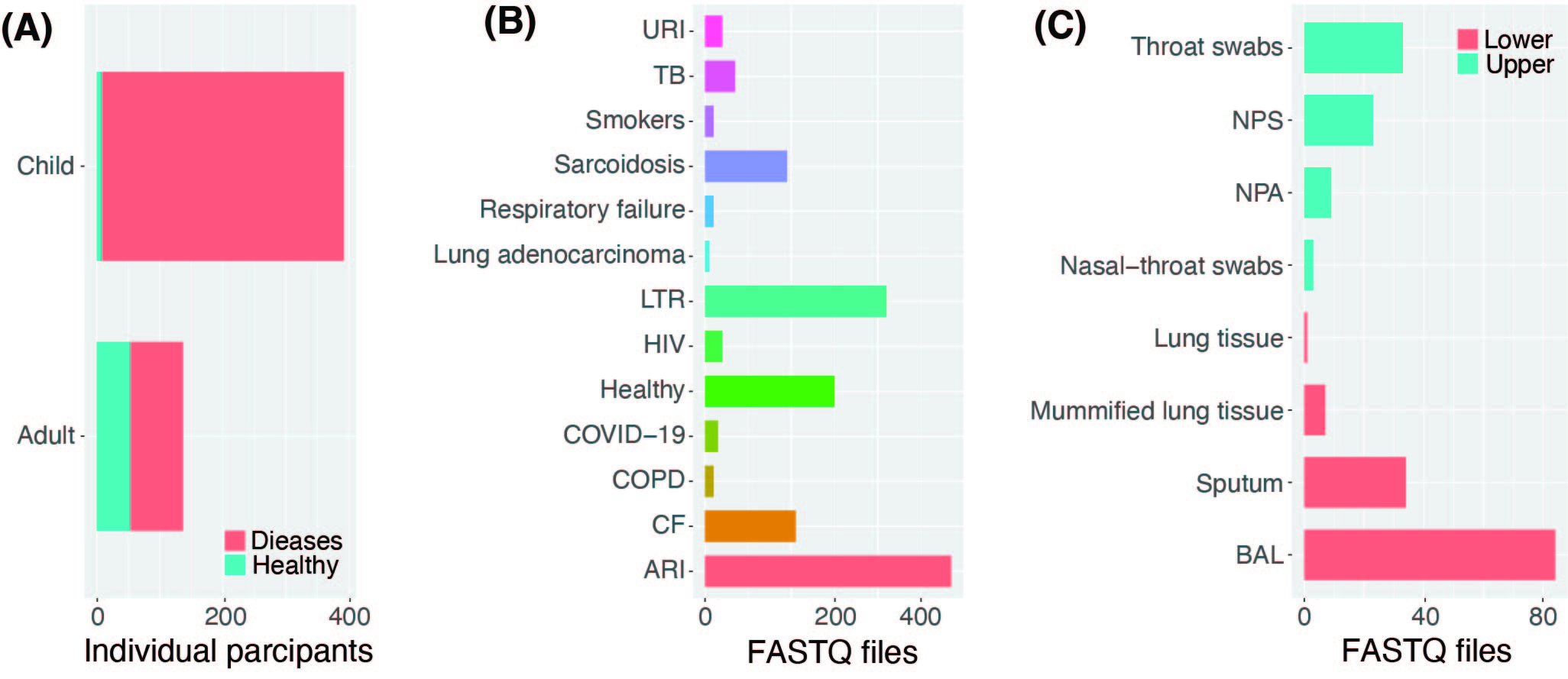


**Supplementary Figure S2.** Demographics of the studies analysed in this study. (A) Stacked bar plot depicts the number of subjects represented in the successful FASTQ files. Study subjects are grouped in two age-related categories; adult and children. For each age group the number of healthy and diseased subjects is depicted in blue and red respectively. (B) Bar plots represent the successful FASTQ files grouped by the clinical phenotype of the subject from whom the associated biological specimen was obtained. (C) Bar plots show the number of FASTQ files grouped by the type of biological specimen from which the sequencing data was generated. Specimens representing the upper and lower airways are indicated in blue and red respectively. *ARI: acute respiratory infection; CF: cystic fibrosis; COPD: chronic obstructive pulmonary disease; HIV: human immunodeficiency virus; LTR: lung transplant recipient; BAL: bronchoalveolar lavage; NPA: nasopharyngeal aspirates; NPS: nasopharyngeal swabs; TB: tuberculosis; URTI: upper respiratory infection.*

**
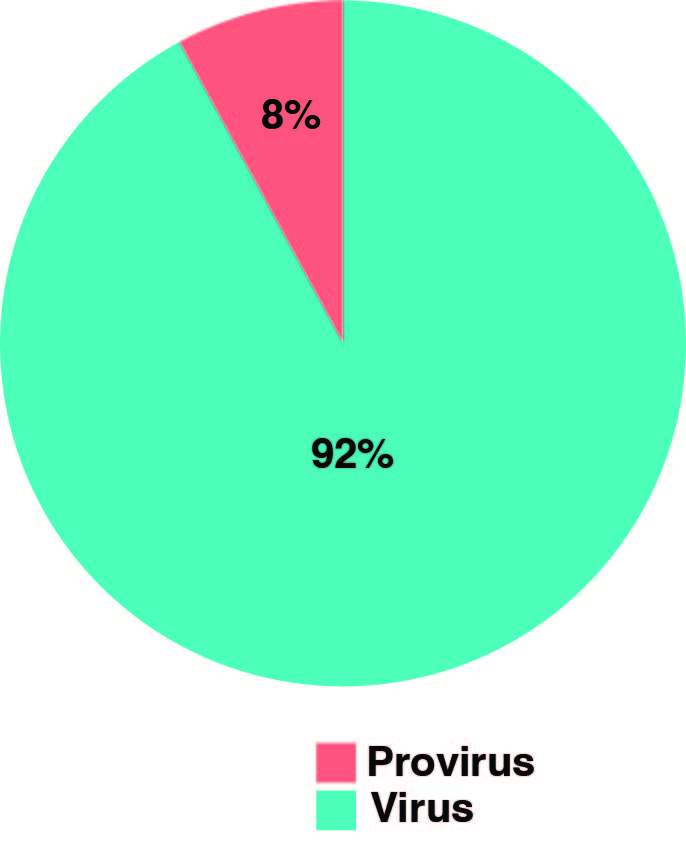
**

**Supplementary Figure S3.** A pie chart showing the distribution of contigs classified as viruses and those as proviruses. Of the 1842 contigs identified, 1696 (92%) were identified as proviruses (red), while 146 (8%) were identified as viruses (blue).


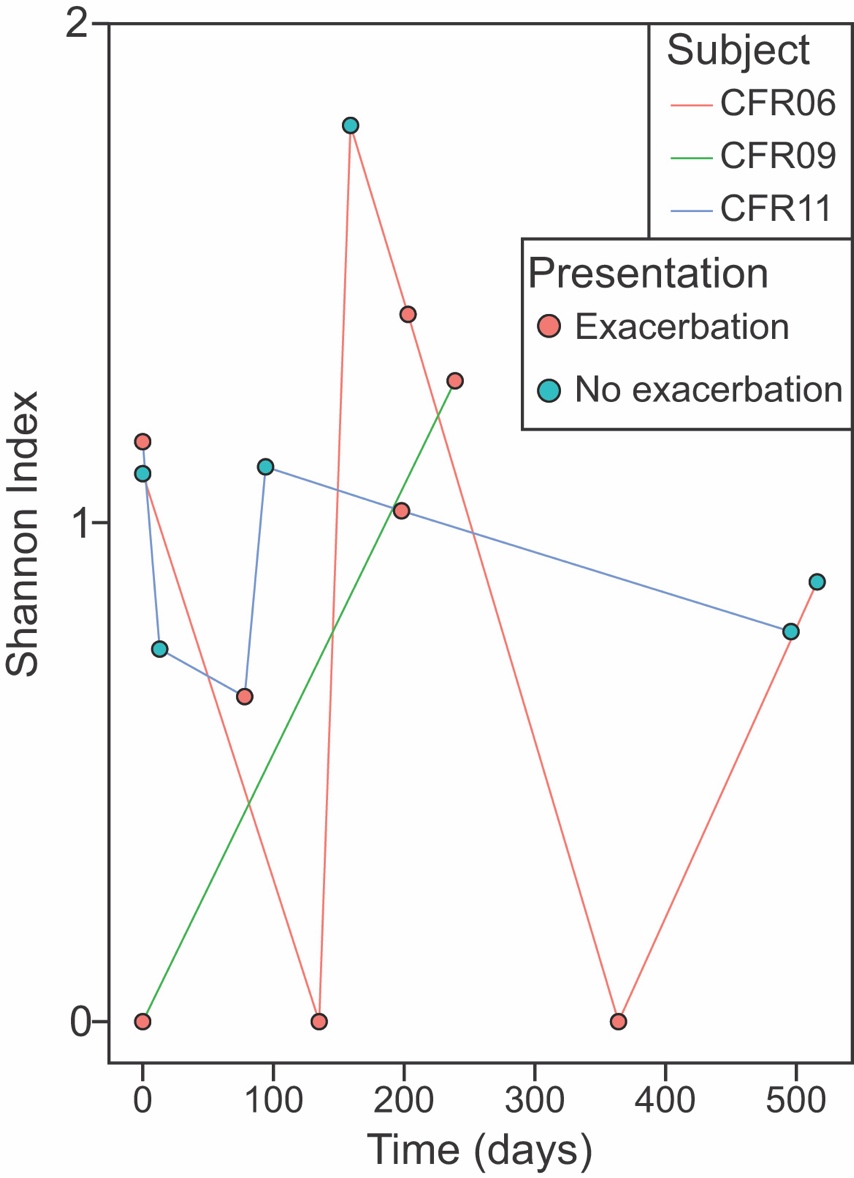


**Supplementary Figure S4.** Scatter plot showing the variation in viral diversity over the time in sputum from 3 patients with cystic fibrosis (bioproject PRJEB32062). Each dot represents an independent sample collected either during an exacerbation episode (red filling) or during a period of clinical stability (blue filling). Samples obtained from the same patient are connected by straigth lines.


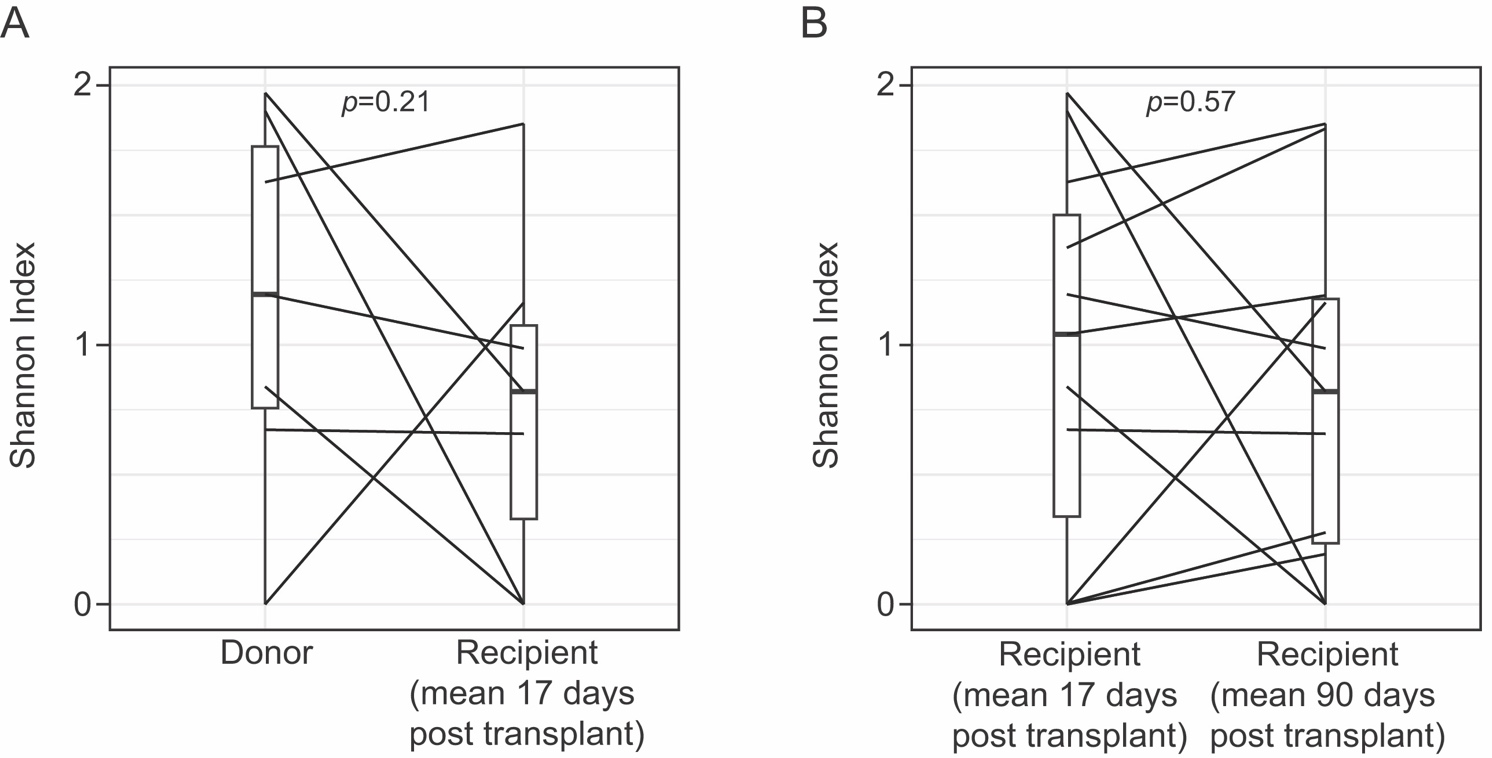


**Supplementary Figure S5.** Boxplots showing alpha diversity estimates (Shannon index) in paired BAL samples. **A**. Comparison between viral diversity in donor lung and the first available sample postransplant (mean 17 days, standard deviation 31 days). **B**. Viral diversity in lung transplant recipients between the first and the second available sample post-transplant (mean 17 days post-transplant, standard deviation 31 days for the first BAL; mean 90 days post-transplant, standard deviation 59 days for the second BAL). Samples collected from the same individual are connected with straight lines. For statistical inference ,we used a paired t-test after confirming normality of data distribution using the Shapiro-Wilk test. The observed p-value is shown in the graph.

**Supplementary Table S1.** Search terms utilized in strategy 1 and 2 for bibliographic data search and data collection.

| **Data mining Search Terms** | |
| --- | --- |
| Strategy 1 search terms | |
| NCBI Bioproject Repository (Mar-Apr 2022) | |
|  | (((Lungs [MeSH Terms]) OR (Respiratory tract [MeSH Terms]) OR (Lungs [Title/Abstract]) OR (“Lower respiratory tract” [Title/Abstract]) OR (“Bronchoalveolar lavage fluid” [MeSH Terms]) OR (Lung sputum [MeSH Terms])) OR (Sputum [Title/Abstract]) OR (“Sputum samples” [Title/Abstract])) AND  ((“Next generation sequencing” [Title/Abstract]) OR (“Shotgun metagenomics” [Title/Abstract]) OR (Metagenomics [Title/Abstract]) OR (Metatranscriptomics [Title/Abstract]) OR (“High throughput sequencing” [Title/Abstract])) AND (((Virome [MeSH Terms]) OR (Viral genome [MeSH Terms])) AND ((Virome [Title/Abstract]) OR (“Lung virome” [Title/Abstract]) OR (“Viral genome” [Title/Abstract]) OR (Bacteriophages [Title/Abstract]) OR (Phages [Title/Abstract]))) NOT ((16s [All Fields]) AND (“Animal models” [All Fields]) AND (Gut [All Fields])) |
| Strategy 2 search terms | |
| NCBI Bioproject Repository (Apr-May 2022) | |
| 1. | Lung virome |
| 2. | Lung virome human |
| 3. | Respiratory virome |
| 4. | Human respiratory virome |
| 5. | Pulmonary virome |
| 6. | Human pulmonary virome |
| 7. | Lung metagenomics |
| 8. | Human lung metagenomics |
| 9. | Lung metatranscriptomics |
| 10. | Human lung metatranscriptomics |

**Supplementary Table S2.** Summary table of the 43 bioprojects and their associated studies, that were mined from literature and sequence databases, with their linked papers, sequencing information, and clinical phenotype metadata summarized. Bioprojects highlighted in blue contain viral contigs identified through EVEREST.

| Bioproject | Article title | Sequencing instrument | Layout | Genomic target | Clinical phenotype | Age | Biological Sample type | Sample size | Reference |
| --- | --- | --- | --- | --- | --- | --- | --- | --- | --- |
| PRJDB9156 | Next-Generation Sequencing Analysis of the Within-Host Genetic Diversity of Influenza A(H1N1)pdm09 Viruses in the Upper and Lower Respiratory Tracts of Patients with Severe Influenza. | Short read: Illumina  Illumina Miseq | Paired (mate sequence) | RNA | Disease: ICU patients with (H1N1)pdm09 | Adult | NPS  Tracheal lavage aspirate | 9 patients | [1] |
| **PRJEB32062** | Strain-Resolved Dynamics of the Lung Microbiome in Patients with Cystic Fibrosis. | Short read: Illumina  Illumina Hiseq  Long read: PacBio  Sequel II | Paired  Single | DNA | Disease: CF | NOT GIVEN: assume adult | Sputum | 11 patients (4 selected for further study as result) | [2] |
| PRJEB41223 | COVID-19 severity and complications associated with low diversity, dysbiosis and predictive metagenome features of the oropharyngeal microbiome. | Short read: Illumina  Illumina Novaseq | Paired | DNA | Disease: COVID19 Also non-COVID but pulmonary disease/respiratory distress | Adult | Oropharyngeal (throat) swabs | Healthy = 74 patients (negative)  URT infection = 112 patients (negative)  Mild Covid = 36 patients (positive)  Mod Covid = 27 patients (positive)  Severe Covid = 66 patients (positive)  315 patients in total | [3] |
| **PRJEB7454** | Eighteenth-century genomes show that mixed infections were common at time of peak tuberculosis in Europe. | Short read: Illumina  Illumina Hiseq | Paired | DNA | Disease: TB | Adult | Lung tissue sample (mummified) | 8 samples in total: 1 from lung, 2 from thorax (though I think these are a no) | [4] |
| PRJNA279445 | Upregulation of Human Endogenous Retrovirus-K Is Linked to Immunity and Inflammation in Pulmonary Arterial Hypertension. | Short read: Illumina  Illumina Miseq | Paired | RNA | Mixed (PAH and health controls - donor samples) | Mixed | Lung tissue sample | 45 patients | [5] |
| PRJNA283035 | Metagenomic analysis of viral genetic diversity in respiratory samples from children with severe acute respiratory infection in China. | Short read: Illumina  Illumina Hiseq | Paired | RNA and DNA | Mixed: disease (SARI), Controls healthy (15) | Children | Nasopharyngeal swabs | 135 Diseased, 15 Healthy | [6] |
| PRJNA316056 | A Different Microbiome Gene Repertoire in the Airways of Cystic Fibrosis Patients with Severe Lung Disease | Short read: Illumina  Illumina Hiseq | Paired | DNA | Disease: CF | Adult | Sputum | 12 patients | [7] |
| **PRJNA316588** | Sputum DNA sequencing in cystic fibrosis: non-invasive access to the lung microbiome and to pathogen details. | Short read: Illumina  Illumina Hiseq | Paired | DNA | Mixed (CF, COPD, smokers, healthy) | Mixed | Sputum | 17 (6 CF, 4 COPD, 7 health - 3 smoke, 4 not) | [8] |
| PRJNA347554 | Reduced biological effect of e-cigarette aerosol compared to cigarette smoke evaluated in vitro using normalized nicotine dose and RNA-seq-based toxicogenomics. | Short read: Illumina  Illumina Hiseq | Paired | RNA | Smokers (e-cig smokers) | Adult | Nasal swab | 3 (non smokers, then used solutions) | [9] |
| **PRJNA369654** | Viral metagenomics reveal blooms of anelloviruses in the respiratory tract of lung transplant recipients. | Short read: Illumina  Illumina Miseq | Paired | RNA and DNA | Mixed (LTRs, HIV and healthy) | Adult | BAL  Oropharyngeal wash | 8 healthy (mix of smoking and non)  3 HIV+ | [10] |
| PRJNA390659 | The Perioperative Lung Transplant Virome: Torque Teno Viruses Are Elevated in Donor Lungs and Show Divergent Dynamics in Primary Graft Dysfunction. | Short read: Illumina  Illumina Hiseq | Paired | RNA and DNA | Disease: LTRs (PGD) | Adult | BAL | 46 samples | [11] |
| **PRJNA392272** | Microbial Lineages in Sarcoidosis. A Metagenomic Analysis Tailored for Low-Microbial Content Samples. | Short read: Illumina  Illumina Hiseq | Paired | RNA and DNA | Mixed (sarcoidosis and healthy controls) | Adult. | BAL | 93 sarcoidosis  72 without sarcoidosis  150 enviromental controls | [12] |
| **PRJNA419524** | Bidirectional transfer of Anelloviridae lineages between graft and host during lung transplantation. | Short read: Illumina  Illumina Hiseq | Paired | RNA and DNA | Mixed (organ transplantation - LTR, and healthy) | Adult | BAL  Serum  (EACH LABELLED) | 13 organ donors/recipient pairs (26)  8 healthy lungs  (114 samples) | [13] |
| PRJNA46335 | The Human Virome in Children and its Relationship to Febrile Illness. | Short read: Illumina  Illumina Genome analyser II | Paired | RNA and DNA | Mixed (fever and no fever, immunocomprimised with fever and immunocomprimised but no fever) | Children | Nasopharyngeal secretions | 178  Specimen collection 228 (may be more) | [14] |
| **PRJNA493096** | Miniaturization and optimization of 384-well compatible RNA sequencing library preparation. | Short read: Illumina  Illumina Hiseq | Paired | RNA | Diseased: pathogens (respiratory) | NOT GIVEN: assume adult | Endotracheal tube aspirate  BAL sample | 2 (from testing method) | [15] |
| **PRJNA494633** | Quality control implementation for universal characterization of DNA and RNA viruses in clinical respiratory samples using single metagenomic next-generation sequencing workflow. | Short read: Illumina  Illumina Nextseq | Paired | RNA and DNA | Diseased (ARI, respiratory conditions - acute respiratory infections) | NOT GIVEN: assume adult | nasopharyngeal swabs (n20)  aspirates (n10)  sputum (n7) | 37 samples (28 positive samples = 6 viral infections, 9 negative samples) | [16] |
| PRJNA516870 | Untargeted Metagenomic Investigation of the Airway Microbiome of Cystic Fibrosis Patients with Moderate-Severe Lung Disease. | Short read: Illumina  Illumina Hiseq | Paired | DNA | Disease: CF (exacerbations and normal), also says mod-severe lung disease (had to be diagnosed with CF) | Mixed | Sputum | 22 patients  79 samples | [17] |
| PRJNA530270 | Effect of Geographic Isolation on the Nasal Virome of Indigenous Children. | Short read: Illumina  Illumina Miseq | Paired | RNA and DNA | Healthy (geographical) | Children | Nasal swab | 63 samples/patients | [18] |
| PRJNA533819 | Lower respiratory tract infections in children requiring mechanical ventilation: a multicentre prospective surveillance study incorporating airway metagenomics. | Short read: Illumina  Illumina Miseq | Paired | RNA | Diseased - critically ill (ARI, respiratory conditions, mechanical ventilation, LRTI) | Children | Tracheal aspirate (culture)  Nasopharyngeal (PCR) | 397 samples/participants | [19] |
| PRJNA560212 | The respiratory virome and exacerbations in patients with chronic obstructive pulmonary disease. | Short read: Illumina  Illumina Nextseq | Paired | RNA and DNA | Disease: COPD (exacerbated) | Adult | Nasopharyngeal swabs | 63 patients (88 swabs) | [20] |
| **PRJNA573045** | Diversity and genomic determinants of the microbiomes associated with COVID-19 and non-COVID respiratory diseases. | Short read: Illumina  Illumina Miseq | Paired  Single | RNA | Mixed (COVID and nonCOVID, may have some other nonCOVID respiratory diseases) | NOT GIVEN: assume adult | Nasopharyngeal swabs  Sputum | 21 RNA-seq metagenomic data(patients/sequences)  11 COVID, 6 COPD, a URTI | [21] |
| **PRJNA601736** | RNA based mNGS approach identifies a novel human coronavirus from two individual pneumonia cases in 2019 Wuhan outbreak. | Short read: Illumina  Illumina Miseq | Paired | RNA | Disease: COVID19 (possibly, showed symptoms) | Adult | BAL | 2 patients (COVID19) | [22] |
| **PRJNA623895** | Rapid metagenomic characterization of a case of imported COVID-19 in Cambodia. | Short read: Illumina  Illumina iSeq | Paired | RNA | Disease: COVID-19 | NOT GIVEN: assume adult | Nasopharyngeal swab  Oropharyngeal swab | 1 patient (First COVID19 patient) | [23] |
| **PRJNA629087** | High-resolution Metatranscriptomic Characterization of the Pulmonary RNA Virome After Lung Transplantation. | Short read: Illumina  Illumina Novaseq | Paired | RNA | Mixed (LTRs, Pulmonary virome after lung transplantation) | NOT GIVEN: assume adult | BAL | 52 LTR patients (mention of 5 per group) | [24] |
| PRJNA634356 | Metagenomic Sequencing To Detect Respiratory Viruses in Persons under Investigation for COVID-19. | Short read: Illumina  Illumina Miseq | Paired  Single | RNA | Mixed (COVID19, non-COVID19, other respiratory related diseases. | Adult | Nasopharyngeal swabs | 75 PUI (patient under investigation) PCR neg PUI (n30), PCR pos PUI (n45) | [25] |
| PRJNA644600 | A hybrid pipeline for reconstruction and analysis of viral genomes at multi-organ level. | Short read: Illumina  Illumina Novaseq | Paired | DNA | Healthy (recently deceased) | NOT GIVEN: assume adult | Lung tissue sample | 1 (deceased individual) | [26] |
| PRJNA671738 | Metatranscriptomics to characterize respiratory virome, microbiome, and host response directly from clinical samples. | Short read: Illumina  Illumina Novaseq | Paired | RNA | Mixed (RSV-ARI and healthy) | Children | Nasal swab | 65 children (22 healthy children) | [27] |
| **PRJNA671740** | Preliminary assessment of viral metagenome from cancer tissue and blood from patients with lung adenocarcinoma. | Short read: Illumina  Illumina Miseq | Paired | RNA and DNA | Disease: lung adenocarcinoma | Adult | Lung tissue sample (also blood but labelled) | 25 patients | [28] |
| PRJNA683885 | Lung microbiome of stable and exacerbated COPD patients in Tshwane, South Africa. | Short read: Illumina  Illumina Miseq | Paired | RNA and DNA | Disease: COPD Stable and exacerbated | Adult | Sputum | 24 patients | [29] |
| **PRJNA779483** | Human viral metagenome Genome sequencing | Short read: Illumina  Illumina Hiseq | Paired | RNA and DNA | Disease: ARI | Children | Throat swabs | 411 children | [30] |
| PRJNA802370 | Multiplexed CRISPR-based microfluidic platform for clinical testing of respiratory viruses and identification of SARS-CoV-2 variants. | Short read: Illumina  Illumina Novaseq | Paired | RNA | Mixed (diseased with respiratory viruses and COVID, also some healthy) | NOT GIVEN: assume adult | Nasopharyngeal swabs | 525 patient specimens | [31] |
| PRJNA744354 | Metagenomic next-generation sequencing to identify pathogens and cancer in lung biopsy tissue. | Short read: Illumina  Illumina Novaseq  Long read: Nanopore  GridION | Paired &  Single | DNA | Disease: pulmonary infection/abnormal chest imaging | Adult | Lung tissue | 133 biopsy samples | [32] |
| **PRJNA189842** | Metagenomic analysis of tuberculosis in a mummy. | Short read: Illumina  Illumina Miseq | Paired | DNA | Disease: TB | Adult | Mummified lung tissue | 1 mummy | [33] |
| PRJEB25186 | Temporal dynamics of the lung and plasma viromes in lung transplant recipients. | Short read: Illumina  Illumina Miseq | Single | RNA and DNA | Disease: LTRs | Adult | BAL  plasma  EACH SAMPLE TYPE LABELLED | 15 LTR recipients  7 patients follow-up samples (24 paired BAL) | [34] |
| PRJEB8320 | The respiratory virome in chronic obstructive pulmonary disease. | Short read: 454 pyrosequencing  GS FLX Titanium | Single | RNA and DNA | Disease: COPD  Stable and exacerbated (2 healthy controls, from database) | Adult | Sputum | 4 patients (10 sputum samples and 2 reference samples) | [35] |
| PRJNA178740 | High-throughput RNA sequencing of a formalin-fixed, paraffin-embedded autopsy lung tissue sample from the 1918 influenza pandemic. | Short read: Illumina  Illumina Genome analyser IIx | Single | RNA | Disease: Influenza | NOT GIVEN: assume adult | Lung tissue FFPE sample | 2 patients (deceased) | [36] |
| PRJNA258008 | Metataxonomic and Metagenomic Approaches vs. Culture-Based Techniques for Clinical Pathology. | Short read: Illumina  Illumina Hiseq | Single | DNA | Disease: drug resistant infections | NOT GIVEN: assume adult | Bronchial aspirates | 8 patients | [37] |
| PRJNA322414 | Metagenome and Metatranscriptome Profiling of Moderate and Severe COPD Sputum in Taiwanese Han Males. | Short read: Illumina  Illumina Miseq | Single &  Paired | RNA and DNA | Disease: COPD (mod - severe, and stable) | Adult | sputum | 8 (4 mod COPD, 4 severe COPD) | [38] |
| PRJNA510441 | Cystic Fibrosis Rapid Response: Translating Multi-omics Data into Clinically Relevant Information. | Short read: Illumina  Illumina Miseq | Single | RNA and DNA | Disease: CF (CFRR - rapid response), abrupt lung function decline | Adult | Sputum | 1 case study patient | [39] |
| PRJNA517266 | A seventeenth-century Mycobacterium tuberculosis genome supports a Neolithic emergence of the Mycobacterium tuberculosis complex. | Short read: Illumina  Illumina Nextseq | Single  Paired | DNA | Disease: TB | Adult | Lung tissue sample (calcified lung nodule) | 1 sample (1 individual) | [40] |
| **PRJNA639353** | The Virome of Acute Respiratory Diseases in Individuals at Risk of Zoonotic Infections. | Short read: Illumina  Illumina Miseq | Single/ Paired | RNA | Disease: respiratory infection/zoonotic | Adult | Nasal/throat swabs | 581 in cohort, 91 with respiratory related disease/symptoms  91 (working with animals), 15 pos (controls) | [41] |
| PRJNA71831 | Metagenomics and metatranscriptomics: windows on CF-associated viral and microbial communities. | Short read: 454  GS-FLX titanium | Single/ Paired | RNA and DNA | Disease: CF | Adult | sputum  tracheal aspirate | 5 patients (12 samples) = 8 samples for virome | [42] |
| PRJNA80601 | Identification of a novel human papillomavirus by metagenomic analysis of samples from patients with febrile respiratory illness. | Short read: 454 pyrosequencing  GS FLX Titanium | Single | RNA and DNA | Mixed (pneumonia, febrile illness, healthy) | Adult | nasopharyngeal swabs  oropharyngeal swabs | 75 samples | [43] |

**Supplemental References**

1. Takayama I, Nguyen BG, Dao CX, Pham TT, Dang TQ, Truong PT, Do T Van, Pham TTP, Fujisaki S, Odagiri T, et al. Next-Generation Sequencing Analysis of the Within-Host Genetic Diversity of Influenza A(H1N1)pdm09 Viruses in the Upper and Lower Respiratory Tracts of Patients with Severe Influenza. mSphere (2021) 6: doi:10.1128/MSPHERE.01043-20

2. Dmitrijeva M, Kahlert CR, Feigelman R, Kleiner RL, Nolte O, Albrich WC, Baty F, von Mering C. Strain-Resolved Dynamics of the Lung Microbiome in Patients with Cystic Fibrosis. mBio (2021) 12:1–20. doi:10.1128/MBIO.02863-20

3. de Castilhos J, Zamir E, Hippchen T, Rohrbach R, Schmidt S, Hengler S, Schumacher H, Neubauer M, Kunz S, Müller-Esch T, Hiergeist A. COVID-19 severity and complications associated with low diversity, dysbiosis and predictive metagenome features of the oropharyngeal microbiome. (2021) doi:10.21203/RS.3.RS-127621/V1

4. Kay GL, Sergeant MJ, Zhou Z, Chan JZM, Millard A, Quick J, Szikossy I, Pap I, Spigelman M, Loman NJ, et al. Eighteenth-century genomes show that mixed infections were common at time of peak tuberculosis in Europe. Nat Commun (2015) 6: doi:10.1038/NCOMMS7717

5. Saito T, Miyagawa K, Chen SY, Tamosiuniene R, Wang L, Sharpe O, Samayoa E, Harada D, Moonen JRAJ, Cao A, et al. Upregulation of Human Endogenous Retrovirus-K Is Linked to Immunity and Inflammation in Pulmonary Arterial Hypertension. Circulation (2017) 136:1920–1935. doi:10.1161/CIRCULATIONAHA.117.027589

6. Wang Y, Zhu N, Li Y, Lu R, Wang H, Liu G, Zou X, Xie Z, Tan W. Metagenomic analysis of viral genetic diversity in respiratory samples from children with severe acute respiratory infection in China. Clin Microbiol Infect (2016) 22:458.e1-458.e9. doi:10.1016/J.CMI.2016.01.006

7. Bacci G, Mengoni A, Fiscarelli E, Segata N, Taccetti G, Dolce D, Paganin P, Morelli P, Tuccio V, De Alessandri A, et al. A Different Microbiome Gene Repertoire in the Airways of Cystic Fibrosis Patients with Severe Lung Disease. Int J Mol Sci (2017) 18: doi:10.3390/IJMS18081654

8. Feigelman R, Kahlert CR, Baty F, Rassouli F, Kleiner RL, Kohler P, Brutsche MH, von Mering C. Sputum DNA sequencing in cystic fibrosis: non-invasive access to the lung microbiome and to pathogen details. Microbiome (2017) 5: doi:10.1186/S40168-017-0234-1

9. Haswell LE, Baxter A, Banerjee A, Verrastro I, Mushonganono J, Adamson J, Thorne D, Gaça M, Minet E. Reduced biological effect of e-cigarette aerosol compared to cigarette smoke evaluated in vitro using normalized nicotine dose and RNA-seq-based toxicogenomics. Sci Rep (2017) 7: doi:10.1038/S41598-017-00852-Y

10. Young JC, Chehoud C, Bittinger K, Bailey A, Diamond JM, Cantu E, Haas AR, Abbas A, Frye L, Christie JD, et al. Viral metagenomics reveal blooms of anelloviruses in the respiratory tract of lung transplant recipients. Am J Transplant (2015) 15:200–209. doi:10.1111/AJT.13031

11. Abbas AA, Diamond JM, Chehoud C, Chang B, Kotzin JJ, Young JC, Imai I, Haas AR, Cantu E, Lederer DJ, et al. The Perioperative Lung Transplant Virome: Torque Teno Viruses Are Elevated in Donor Lungs and Show Divergent Dynamics in Primary Graft Dysfunction. Am J Transplant (2017) 17:1313–1324. doi:10.1111/AJT.14076

12. Clarke EL, Lauder AP, Hofstaedter CE, Hwang Y, Fitzgerald AS, Imai I, Biernat W, Rȩkawiecki B, Majewska H, Dubaniewicz A, et al. Microbial Lineages in Sarcoidosis. A Metagenomic Analysis Tailored for Low-Microbial Content Samples. Am J Respir Crit Care Med (2018) 197:225–234. doi:10.1164/RCCM.201705-0891OC

13. Abbas AA, Young JC, Clarke EL, Diamond JM, Imai I, Haas AR, Cantu E, Lederer DJ, Meyer K, Milewski RK, et al. Bidirectional transfer of anelloviridae lineages between graft and host during lung transplantation. Am J Transplant (2019) 19:1086. doi:10.1111/AJT.15116

14. Jaffe D, Muenzer J, Storch G, Weinstock G, Sodergren E, Wylie K, Arens M, Buller R. The Human Virome in Children and its Relationship to Febrile Illness. Nature Precedings 2010 (2010)1–1. doi:10.1038/npre.2010.5319.1

15. Mayday MY, Khan LM, Chow ED, Zinter MS, DeRisi JL. Miniaturization and optimization of 384-well compatible RNA sequencing library preparation. PLoS One (2019) 14:e0206194. doi:10.1371/JOURNAL.PONE.0206194

16. Bal A, Pichon M, Picard C, Casalegno JS, Valette M, Schuffenecker I, Billard L, Vallet S, Vilchez G, Cheynet V, et al. Quality control implementation for universal characterization of DNA and RNA viruses in clinical respiratory samples using single metagenomic next-generation sequencing workflow. BMC Infect Dis (2018) 18: doi:10.1186/S12879-018-3446-5

17. Bacci G, Taccetti G, Dolce D, Armanini F, Segata N, Di Cesare F, Lucidi V, Fiscarelli E, Morelli P, Casciaro R, et al. Untargeted Metagenomic Investigation of the Airway Microbiome of Cystic Fibrosis Patients with Moderate-Severe Lung Disease. Microorganisms (2020) 8:1–18. doi:10.3390/MICROORGANISMS8071003

18. Altan E, Dib JC, Gulloso AR, Juandigua DE, Deng X, Bruhn R, Hildebrand K, Freiden P, Yamamoto J, Schultz-Cherry S, et al. Effect of Geographic Isolation on the Nasal Virome of Indigenous Children. J Virol (2019) 93: doi:10.1128/JVI.00681-19

19. Tsitsiklis A, Osborne CM, Kamm J, Williamson K, Kalantar K, Dudas G, Caldera S, Lyden A, Tan M, Neff N, et al. Lower respiratory tract infections in children requiring mechanical ventilation: a multicentre prospective surveillance study incorporating airway metagenomics. Lancet Microbe (2022) 3:e284–e293. doi:10.1016/S2666-5247(21)00304-9

20. Van Rijn AL, Van Boheemen S, Sidorov I, Carbo EC, Pappas N, Mei H, Feltkamp M, Aanerud M, Bakke P, Claas ECJ, et al. The respiratory virome and exacerbations in patients with chronic obstructive pulmonary disease. PLoS One (2019) 14:e0223952. doi:10.1371/JOURNAL.PONE.0223952

21. Hoque MN, Rahman MS, Ahmed R, Hossain MS, Islam MS, Islam T, Hossain MA, Siddiki AZ. Diversity and genomic determinants of the microbiomes associated with COVID-19 and non-COVID respiratory diseases. Gene Rep (2021) 23: doi:10.1016/J.GENREP.2021.101200

22. Chen L, Liu W, Zhang Q, Xu K, Ye G, Wu W, Sun Z, Liu F, Wu K, Zhong B, et al. RNA based mNGS approach identifies a novel human coronavirus from two individual pneumonia cases in 2019 Wuhan outbreak. Emerg Microbes Infect (2020) 9:313–319. doi:10.1080/22221751.2020.1725399

23. Manning JE, Bohl JA, Lay S, Chea S, Sovann L, Sengdoeurn Y, Heng S, Vuthy C, Kalantar K, Ahyong V, et al. Rapid metagenomic characterization of a case of imported COVID-19 in Cambodia. bioRxiv (2020) doi:10.1101/2020.03.02.968818

24. Mitchell AB, Li CX, Oliver BGG, Holmes EC, Glanville AR. High-resolution Metatranscriptomic Characterization of the Pulmonary RNA Virome After Lung Transplantation. Transplantation (2021) 105:2546–2553. doi:10.1097/TP.0000000000003713

25. Babiker A, Bradley HL, Stittleburg VD, Ingersoll JM, Key A, Kraft CS, Waggoner JJ, Piantadosi A. Metagenomic Sequencing To Detect Respiratory Viruses in Persons under Investigation for COVID-19. J Clin Microbiol (2020) 59: doi:10.1128/JCM.02142-20

26. Pratas D, Toppinen M, Pyoria L, Hedman K, Sajantila A, Perdomo MF. A hybrid pipeline for reconstruction and analysis of viral genomes at multi-organ level. Gigascience (2020) 9: doi:10.1093/GIGASCIENCE/GIAA086

27. Rajagopala S V., Bakhoum NG, Pakala SB, Shilts MH, Rosas-Salazar C, Mai A, Boone HH, McHenry R, Yooseph S, Halasa N, et al. Metatranscriptomics to characterize respiratory virome, microbiome, and host response directly from clinical samples. Cell reports methods (2021) 1: doi:10.1016/J.CRMETH.2021.100091

28. Cai HZ, Zhang H, Yang J, Zeng J, Wang H. Preliminary assessment of viral metagenome from cancer tissue and blood from patients with lung adenocarcinoma. J Med Virol (2021) 93:5126–5133. doi:10.1002/JMV.26887

29. Goolam Mahomed T, Peters RPH, Allam M, Ismail A, Mtshali S, Goolam Mahomed A, Ueckermann V, Kock MM, Ehlers MM. Lung microbiome of stable and exacerbated COPD patients in Tshwane, South Africa. Sci Rep (2021) 11: doi:10.1038/S41598-021-99127-W

30. Mao Q, Sun G, Qian Y, Qian Y, Li W, Wang X, Shen Q, Yang S, Zhou C, Wang H, Zhang W. Viral metagenomics of pharyngeal secretions from children with acute respiratory diseases with unknown etiology revealed diverse viruses. Virus Res. (2022) 321:198912. doi: 10.1016/j.virusres.2022.198912. Epub 2022 Sep 2. PMID: 36058285.

31. Welch NL, Zhu M, Hua C, Weller J, Mirhashemi ME, Nguyen TG, Mantena S, Bauer MR, Shaw BM, Ackerman CM, et al. Multiplexed CRISPR-based microfluidic platform for clinical testing of respiratory viruses and identification of SARS-CoV-2 variants. Nature Medicine 2022 28:5 (2022) 28:1083–1094. doi:10.1038/s41591-022-01734-1

32. Guo Y, Li H, Chen H, Li Z, Ding W, Wang J, Yin Y, Jin L, Sun S, Jing C, et al. Metagenomic next-generation sequencing to identify pathogens and cancer in lung biopsy tissue. EBioMedicine (2021) 73: doi:10.1016/J.EBIOM.2021.103639

33. Chan JZ-M, Sergeant MJ, Lee OY-C, Minnikin DE, Besra GS, Pap I, Spigelman M, Donoghue HD, Pallen MJ. Metagenomic analysis of tuberculosis in a mummy. N Engl J Med (2013) 369:289–290. doi:10.1056/NEJMC1302295

34. Segura-Wang M, Görzer I, Jaksch P, Puchhammer-Stöckl E. Temporal dynamics of the lung and plasma viromes in lung transplant recipients. PLoS One (2018) 13:e0200428. doi:10.1371/JOURNAL.PONE.0200428

35. Garcia-Nuñez M, Gallego M, Monton C, Capilla S, Millares L, Pomares X, Espasa M, Ferrari R, Moya A, Monsó E, et al. The respiratory virome in chronic obstructive pulmonary disease. Future Virol (2018) 13:457–466. doi:10.2217/FVL-2018-0027

36. Xiao YL, Kash JC, Beres SB, Sheng ZM, Musser JM, Taubenberger JK. High-throughput RNA sequencing of a formalin-fixed, paraffin-embedded autopsy lung tissue sample from the 1918 influenza pandemic. J Pathol (2013) 229:535–545. doi:10.1002/PATH.4145

37. Hilton SK, Castro-Nallar E, Pérez-Losada M, Toma I, McCaffrey TA, Hoffman EP, Siegel MO, Simon GL, Johnson WE, Crandall KA. Metataxonomic and Metagenomic Approaches vs. Culture-Based Techniques for Clinical Pathology. Front Microbiol (2016) 7: doi:10.3389/FMICB.2016.00484

38. Lee SW, Kuan CS, Wu LSH, Weng JTY. Metagenome and Metatranscriptome Profiling of Moderate and Severe COPD Sputum in Taiwanese Han Males. PLoS One (2016) 11:e0159066. doi:10.1371/JOURNAL.PONE.0159066

39. Güemes AGC, Lim YW, Quinn RA, Conrad DJ, Benler S, Maughan H, Edwards R, Brettin T, Cantú VA, Cuevas D, et al. Cystic Fibrosis Rapid Response: Translating Multi-omics Data into Clinically Relevant Information. mBio (2019) 10: doi:10.1128/MBIO.00431-19

40. Sabin S, Herbig A, Vågene ÅJ, Ahlström T, Bozovic G, Arcini C, Kühnert D, Bos KI. A seventeenth-century Mycobacterium tuberculosis genome supports a Neolithic emergence of the Mycobacterium tuberculosis complex. Genome Biol (2020) 21: doi:10.1186/S13059-020-02112-1

41. Kha Tu NT, Thu Hong NT, Han Ny NT, Phuc TM, Thanh Tam PT, van Doorn HR, Trung Nghia HD, Huong DT, Han DA, Thu Ha LT, et al. The Virome of Acute Respiratory Diseases in Individuals at Risk of Zoonotic Infections. Viruses (2020) 12: doi:10.3390/V12090960

42. Lim YW, Schmieder R, Haynes M, Willner D, Furlan M, Youle M, Abbott K, Edwards R, Evangelista J, Conrad D, et al. Metagenomics and metatranscriptomics: windows on CF-associated viral and microbial communities. J Cyst Fibros (2013) 12:154–164. doi:10.1016/J.JCF.2012.07.009

43. Mokili JL, Dutilh BE, Lim YW, Schneider BS, Taylor T, Haynes MR, Metzgar D, Myers CA, Blair PJ, Nosrat B, et al. Identification of a novel human papillomavirus by metagenomic analysis of samples from patients with febrile respiratory illness. PLoS One (2013) 8: doi:10.1371/JOURNAL.PONE.0058404
